# Evaluation of the Utility of Point-of-Care HIV Testing on a Canadian Internal Medicine Inpatient Unit

**DOI:** 10.1155/2017/8495307

**Published:** 2017-11-02

**Authors:** Lawrence Lau, Beverly Wudel, Eugene Lee, Majid Darraj, Quinlan Richert, Adriana Trajtman, Kim Bresler, Jared Bullard, Ken Kasper, Marissa Becker, Yoav Keynan

**Affiliations:** ^1^Department of Internal Medicine, Rady Faculty of Health Sciences, University of Manitoba, Winnipeg, MB, Canada; ^2^Department of Medical Microbiology and Infectious Diseases, Rady Faculty of Health Sciences, University of Manitoba, Winnipeg, MB, Canada; ^3^Department of Internal Medicine, Jazan University, Jazan, Saudi Arabia; ^4^Manitoba HIV Program, Health Sciences Centre, Winnipeg, MB, Canada; ^5^Department of Pediatrics and Child Health, Rady Faculty of Health Sciences, University of Manitoba, Winnipeg, MB, Canada; ^6^Cadham Provincial Laboratory, Winnipeg, MB, Canada; ^7^Centre for Global Public Health, University of Manitoba, Winnipeg, MB, Canada

## Abstract

Point-of-care (POC) HIV testing has been shown to be an acceptable method for increasing HIV testing uptake. To date, no studies have examined the use of POC testing for routine HIV screening on the medicine inpatient unit. A prospective cross-sectional study was conducted over a three-month period in July, August, and October 2016 to evaluate the prevalence of undiagnosed HIV and the attitudes towards routine POC HIV testing. Patients admitted directly to medicine inpatient teaching units at a tertiary hospital in Winnipeg, Canada, were approached for participation. The POC HIV test was administered at the bedside. Reactive and indeterminate tests were confirmed with standard serological HIV testing. Participants were given a questionnaire regarding their attitudes towards POC testing on the unit. Although no cases of previously undiagnosed HIV were identified during the study period, only 35% of participants were found to have ever had HIV testing previously. The majority of participants were satisfied with the POC testing experience and would choose to have the POC testing again. Overall, the low rate of outpatient testing highlights the need for routine HIV testing on an inpatient basis.

## 1. Introduction

In Canada, an estimated 75,500 people were living with HIV by the end of 2014 [1]. Undiagnosed HIV represents a serious public health challenge, with about 21% of infected individuals unaware of their status [1]. Early recognition and initiation of antiretroviral therapy has been shown to reduce transmission and morbidity, and ultimately improve quality of life [2–4]. Accordingly, Health Canada recommends consideration of routine HIV screening during medical care [5].

Individuals who are newly diagnosed with HIV in Manitoba are immediately linked to the Manitoba HIV Program, which provides specialized and long-term care to all individuals living with HIV in the province. In Manitoba, there were 79,924 tests performed, of which 135 (0.17%) were positive; 68,026 unique individuals were tested, of which 134 (0.20%) were positive. There were 78 cases of previously undiagnosed HIV identified in 2015, representing a crude rate of 8.0 cases per 100,000 population. A large percentage (30%) of these cases presented as late diagnoses, with CD4 counts below 200 cells/ml, signifying advanced disease associated with high morbidity and healthcare-related costs [6]. Furthermore, late diagnosis contributes to transmission of undiagnosed HIV to sexual partners. The high proportion of late diagnoses highlights the need for earlier diagnosis through routine screening [7].

Point-of-care (POC) HIV testing has been implemented as a method for overcoming some of the patient and provider barriers to routine screening. POC HIV testing has been shown to be an acceptable method for increasing HIV testing uptake, by providing immediate results, decreasing turnaround time, and guiding urgent decision-making [8]. The INSTI HIV-1/HIV-2 Antibody Test has been validated and licensed for use by Health Canada since 2005 [9]. In 2010, the British Columbia Centre for Disease Control (BCCDC) introduced a centralized POC HIV testing program within the province to expand the availability of HIV testing. BCCDC guidelines suggest POC HIV testing in clinical scenarios where there is an urgent need to determine HIV status, including in acutely ill individuals or in settings in which the prevalence of HIV is expected to be high [10]. Several studies examining the use of POC HIV testing in emergency departments of tertiary care centres have found a prevalence of undiagnosed HIV between 0% and 1.4% [8, 11–13]. Evidence suggests that the prevalence of undiagnosed HIV may be comparable on hospital inpatient wards [14].

The aim of this study was to evaluate the prevalence of previous outpatient HIV testing, the prevalence of undiagnosed HIV, and the attitudes towards routine POC HIV testing in patients admitted to the adult medicine inpatient unit, thereby informing the expansion of HIV testing in the province.

## 2. Methods

### 2.1. Study Design

A prospective cross-sectional study was conducted on patients admitted to two adult medicine inpatient units at Health Sciences Centre (HSC) in Winnipeg, Manitoba, during the months of July, August, and October, 2016. Data was not collected in September due to the unavailability of study staff during this month. HSC is an 850-bed tertiary care centre located in inner-city Winnipeg that provides services to rural Manitoba, Nunavut, and parts of Northwestern Ontario. All patients admitted directly to two adult medicine inpatient teaching units at HSC were approached for participation in the study. Exclusion criteria included known HIV-positive status, disturbed cognition, inability to provide consent, and if the patient declined to be tested for HIV. Ethics approval was obtained from the Health Research Ethics Board of the University of Manitoba.

### 2.2. Data Collection

Study staff was trained on the use of POC HIV test (INSTI HIV-1/HIV-2 Antibody Test, bioLytical Labs, Canada) and standardized pre- and posttest counselling prior to the study. Pretest HIV counselling included information about modes of transmission, risks of infection, diagnosis, the Manitoba HIV Program, and an explanation of the POC HIV test. Posttest counselling was tailored to the POC HIV test result and provided information about the window period and recommendations for future testing. After admission to the inpatient unit, patients were approached by the study staff for participation and consent. The time elapsed between admission and test administration ranged from 0 to 3 days. After obtaining written informed consent, pretest counselling was provided. A brief questionnaire was administered, which collected information on demographics, reason for admission to hospital, previous HIV testing, and risk factors for HIV. The POC HIV test was then performed at the bedside, and results were recorded and shared with the patient immediately. A venous blood sample was drawn from patients who had either a reactive or an indeterminate POC HIV test and sent to Cadham Provincial Laboratory (Winnipeg, Manitoba) for confirmatory testing, consisting of an immunoassay targeted to detect HIV-1/-2 antibodies and p24 antigen. In the event of a positive confirmatory test, patients were to be linked to the Manitoba HIV Program for further interdisciplinary management of newly diagnosed HIV. Posttest counselling was administered, which included information regarding the importance of retesting and avoidance of high-risk behaviours. Afterwards, patients were provided a posttest questionnaire to evaluate their degree of satisfaction with the POC HIV test.

### 2.3. Data Analysis

Descriptive statistics were used to represent data. Data were summarized using percentages and medians with interquartile ranges.

## 3. Results

### 3.1. Patient Recruitment

**Figure 1:**
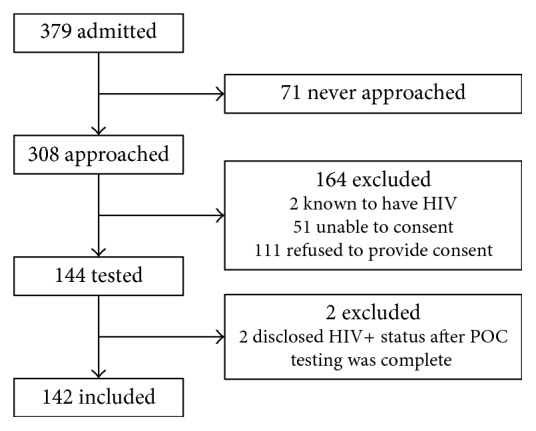
Study design and patient recruitment.

A total of 379 patients were admitted to the adult medicine inpatient teaching units during the study period. Of these patients, 308 were approached for participation in the study, of which 144 ultimately participated in the study and had a POC HIV test (Figure 1). A total of 71 patients were never approached for consent to participate in the study because they were either receiving medical care, were having investigations, or were unwilling to be approached by study staff. A total of 164 patients were excluded due to reasons listed in Table 1. Another two patients were excluded following the administration of POC HIV testing because they disclosed their previously known HIV positive status after testing was performed. The remaining 142 patients were included in analysis.

### 3.2. Baseline Characteristics

Baseline characteristics of the study population are displayed in Table 2. The median age of study participants was 58 (42–68) years, with 68 (48%) males, 73 (51%) females, and 1 (1%) who identified as transgender. The majority of participants self-identified as Caucasian (57%) or Indigenous, including First Nations, Inuit, and Métis (36%), and most (89%) participants were born in Canada. At the time of study enrolment, 98 (69%) listed their primary location of residence to be within Winnipeg, 19 (13%) lived outside Winnipeg in another community within Manitoba, 18 (13%) lived within a First Nation Reserve, and 7 (4%) lived outside Manitoba.

A total of 55 (39%) participants listed the reason for admission as primarily related to an infectious process. A total of 111 participants reported having a regular primary care provider, of whom 39 (35%) reported ever having been tested for HIV previously. Of the 106 participants who had seen their primary care provider within the last year, 7 (7%) reported being tested for HIV during that time.

### 3.3. POC HIV Testing Results and Patient Acceptance

A total of 138 (97%) participants had a negative POC HIV test result, 1 (1%) had a reactive result, and 3 (2%) had an indeterminate result. All participants who tested reactive or indeterminate were subsequently tested negative with routine serologic testing. The results of the posttest questionnaire are shown in Table 3. Of the participants who underwent POC HIV testing, 131 (92%) reported satisfaction with the testing experience and 123 (87%) would choose to have the POC method of testing again if repeat HIV testing was required.

## 4. Discussion

Health Canada recommends that consideration of HIV testing be made a component of routine medical care [5]. In 2014, the Joint United Nations Programme on HIV/AIDS (UNAIDS) launched a strategy to improve the uptake, quality, and outcome of antiretroviral therapy for HIV worldwide, called the “90-90-90” campaign. This campaign aims to have 90% who have HIV diagnosed, 90% of those diagnosed to receive sustained antiretroviral therapy, and 90% of those on therapy to achieve viral suppression [15]. In our study, only 7% of individuals who had been seen by their family physician in the previous year had HIV testing performed during that year. This low rate of testing is consistent with provincial data suggesting that approximately 6% of adults aged 15 years and above receive HIV testing. The fact that only 35% indicated to ever having been tested further illustrates that HIV testing is suboptimal in Manitoba, Canada. The low rate of routine HIV testing reflects a high rate of missed opportunities: instances where HIV-infected individuals present to medical care with possible HIV-related illnesses, but who are not tested for HIV. The prevalence of missed opportunities is generally high across various populations [14, 16–19]. Inpatient units provide a unique opportunity for HIV recognition and linkage to long-term follow-up care. A study by Rucker et al. conducted at three hospitals in Chicago, Illinois, found that inpatient areas had the highest seroprevalence of undiagnosed HIV (0.6%) compared to emergency room (0.4%) and outpatient care areas (0.1%), highlighting the need to focus on inpatient HIV screening [14].

In our study, participant satisfaction with POC HIV testing was high, with 93% of respondents indicating that they were satisfied with the overall testing experience. This is consistent with previous studies showing similar degrees of overall satisfaction with POC testing [8, 20]. A clear majority (87%) of participants indicated that they would choose to have the POC HIV test performed in lieu of the standard HIV test. This falls in line with previous studies that have demonstrated 40–90% of patients prefer rapid POC testing as opposed to standard testing [21–24].

The present study did not detect any undiagnosed HIV on the inpatient units during the study period; however, there continues to be a role for routine inpatient HIV screening. Among several urban tertiary care centres in the United States, the prevalence of undiagnosed HIV on inpatient hospital units is highly variable. This variability appears to be reflective of the study sample size: in the study by Padrnos and colleagues, 283 patients were screened with a reported prevalence of undiagnosed HIV of 0%; Osorio and colleagues screened 1537 patients and reported a prevalence of 0.4%; and finally, the group of Rucker and colleagues screened 7546 patients with a prevalence of 0.6% [14, 25, 26]. Additionally, the failure to identify HIV in an inpatient hospital setting—where a considerable population of patients receive the majority of their medical care—would constitute a critical missed opportunity. This may contribute to the finding that patients diagnosed with HIV while admitted to an inpatient hospital unit tend to have more advanced disease compared to patients diagnosed with HIV in an outpatient setting [27]. Considering that there are few existing studies specifically addressing inpatient HIV prevalence, the finding of no cases of undiagnosed HIV in this present study of relatively small sample size should not dismiss the importance of inpatient screening. Further study to specifically address the prevalence of HIV on an inpatient unit is required.

Overall, POC HIV testing is relatively simple to administer on the medicine inpatient unit and requires minimal prerequisite training. The method of serum acquisition by fine-needle finger poke provides patients and their care providers with a rapid and accurate result through comparatively minimally invasive means. Comparing the cost of one POC test ($15.57 CAD) and the cost of running one ELISA for HIV-1/2 and p24 antigen ($10.72 CAD), the cost-effectiveness of implementing routine POC testing would not be derived from testing alone, but rather, by reducing impending healthcare costs on the burden of illness in patients living with undiagnosed HIV. In practice, POC HIV testing may be particularly useful in settings where knowing HIV status changes the selection of empiric antibiotic coverage, in very short inpatient admissions or in patients with decreased access to services in rural or remote regions.

There are several limitations in this study. First, the opt-in nature of the study design resulted in a significant number of patients who declined POC HIV testing, which may have contributed to selection bias towards patients who were at low risk of having HIV. Previous studies also show low rates of HIV testing uptake in patients offered HIV POC testing by an opt-in approach [28–30]. Reasons for patient refusal of HIV testing include recent testing outside of the study, lack of perceived risk, desire to focus on the primary reason for the visit, fear of psychosocial consequences of diagnosis, and concerns about confidentiality following a diagnosis [20, 31]. It has been shown that patients who decline HIV testing may be at higher risk than those who accept testing [32, 33]. Information that may have provided further insight into reasons for testing refusal, such as demographics, HIV risk factors, and previous HIV testing, was not collected from patients who refused to participate in the study. Without this information, there can only be speculation as to how the collected data may have been biased towards having tested patients at lower risk of having HIV in our study sample. Second, three participants had either indeterminate or falsely positive POC HIV testing results. The sensitivity and specificity of the bioLytical INSTI HIV-1/HIV-2 antibody tests have been reported to be 99–100% and 99%, respectively, based on previously published validation studies [34, 35]. As the objective of this study was not to validate the intrinsic accuracy of this test, there can be no conclusions made from the results about this aspect of the test. However, it is well known that the positive predictive value of the test is dependent upon the pretest probability and population prevalence of HIV. It is, therefore, possible that the aforementioned selection bias for low-risk individuals influences the positive predictive value of the POC HIV test in this study.

## 5. Conclusions

POC HIV testing is well-accepted among patients who receive it and can be used to be used in conjunction with other strategies to overcome the barriers to HIV testing. This study did not identify cases of undiagnosed HIV. However, the sample size was small and may have been biased towards testing lower-risk patients given the opt-in nature of performing POC testing. The proportion of results that were falsely positive or indeterminate may have been anomalously high when compared to previously reported data regarding the sensitivity and specificity of the POC test. This may relate to the overall low prevalence of HIV on the inpatient units we studied; however, unfortunately, this can only be speculated as we did not collect data on the group that refused to participate. Nevertheless, there was a favourable attitude towards the POC HIV test among patients who agreed to be tested, and the test itself was simple and unobtrusive to perform on the medicine unit. Further study utilizing an opt-out approach to testing may more accurately characterize the prevalence of undiagnosed HIV on the adult internal medicine inpatient unit. It has been shown that POC HIV testing would be a feasible method of approaching a screening initiative of this kind and may also capture important data missing from this present study.

## Figures and Tables

**Table 1:** Reasons for patient exclusion from study participation (*n* = 164).

Reason	Prevalence
Refused to participate in study (too tired, too ill, etc.)	58 (35%)
Did not want to know HIV status	7 (4.3%)
Perceived to lack risk	6 (3.6%)
Palliative	3 (1.8%)
No reason given	37 (23%)
Unable to consent	51 (31%)
Already known to have HIV	2 (1.2%)

**Table 2:** Self-reported patient baseline demographics (*n* = 142).

	*n* (%)
*Gender*	
Male	68 (48%)
Female	73 (51%)
Transgender	1 (1%)
*Primary location of residence*	
Winnipeg	98 (68%)
Rural Manitoba, nonreserve	18 (12%)
First Nation Reserve	19 (15%)
Outside Manitoba	7 (4%)
*Ethnicity*	
Caucasian	77 (54%)
First Nation, Métis, or Inuit	52 (37%)
Asian	4 (3%)
African Canadian	2 (1%)
Latin American	1 (1%)
Arab, West Asian, South	
Asian and others	6 (4%)
*Country of birth*	
Canada	126 (89%)
Outside of Canada	16 (11%)
Europe	6 (4%)
South America	4 (3%)
West Pacific	4 (3%)
Africa	1 (1%)
Unknown	1 (1%)

**Table 3:** Results of post-POC testing questionnaire regarding attitudes towards POC HIV test.

Aspect of POC HIV test on questionnaire	Strongly agree or agree, *n* (%)	Neutral, disagree, or strongly disagree, *n* (%)	No response, *n* (%)
Was satisfied with the POC HIV testing experience	131 (92%)	9 (6%)	2 (1%)
Would choose to have a POC HIV test again	123 (87%)	18 (13%)	1 (1%)
Felt anxious during the POC HIV test	35 (25%)	107 (75%)	0 (0%)
